# Developments in Stereotactic Body Radiotherapy

**DOI:** 10.3390/cancers10120497

**Published:** 2018-12-07

**Authors:** Anoop Haridass

**Affiliations:** Clatterbridge Cancer Center, Wirral CH63 4JY, UK; anoopharidass1@nhs.net; Tel.: +44-0151-556-5723

**Keywords:** stereotactic radiotherapy, SBRT, SABR, oligometastases, cancer

## Abstract

Stereotactic body radiotherapy is the technique of accurately delivering high doses of radiotherapy to small volume targets in a single or small number of sessions. The high biological effective dose of this treatment is reflected in the high rates of local control achieved across multiple tumour sites. Toxicity of the treatment can be significant and ongoing prospective trials will help define the utility of this treatment as an alternative to surgery in treating primary tumours and oligometastatic disease. Longer follow-up and survival data from prospective trials will be essential in determining the value of this resource-intensive treatment. The opportunity to combine this treatment with systemic therapies and its potential synergy with immunotherapy opens up interesting avenues for research in the future.

## 1. Introduction

Stereotactic Body Radiotherapy (SBRT) is a method of delivering a high dose of external beam radiotherapy (RT) very precisely to small extra cranial tumour targets, in a small number of session (fractions) with high levels of targeting accuracy [1]. The term SABR (Stereotactic Ablative Body Radiotherapy) has been interchangeably used with SBRT, due to the potential of these high dose-per-session (hypofractionated) treatments to effectively ‘ablate’ the tumour by delivering a high biologically effective dose, although this is still an area of continuing debate [2]. Over the last two decades, rapid technological evolution in radiotherapy delivery, imaging, and computing has made this technique accessible to patients in more radiotherapy treatment centers. 

## 2. Background

**Figure 1:**
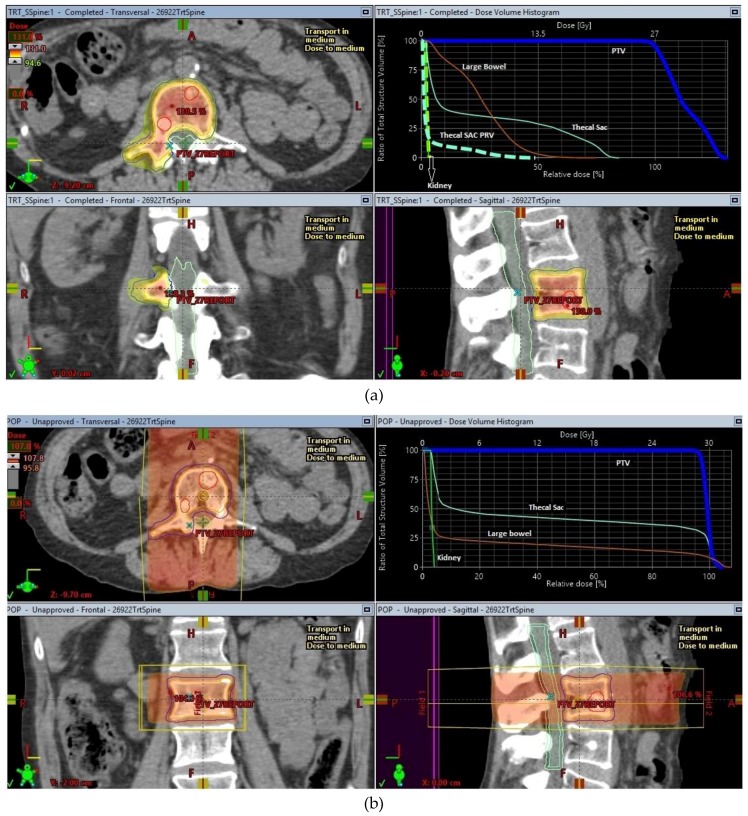
SBRT and conventional treatment plans for a lumbar spine oligometastases. (**a**) SBRT plan for lumbar spine oligometastasis, (**b**) Conventional radiotherapy plan for lumbar spine oligometastasis.

The origins of SBRT are linked to intracranial stereotactic radiosurgery, a concept pioneered and carried forward to full clinical execution by Prof. Lars Leksell [3] with the creation of specialized radiosurgery machines like the Gamma knife^®^ to treat brain tumours. Early work by Lax and Blomgren at the Karolinska Hospital in Stockholm helped to establish the feasibility of high-dose radiotherapy treatments in the extracranial setting [4,5], using stereotactic body frames for achieving the required degree of accuracy that these treatments required. As radiosurgery started to become an established method of treating intracranial lesions, it led to more research into making radiosurgery less invasive. This in turn helped to drive continuing improvements in the delivery of this treatment using more common radiotherapy delivery platforms like linear accelerators. An illustration of the conformal dose distribution achievable with SBRT in comparison to conventional RT for a spinal metastasis is shown in Figure 1. 

Rapid advances in the technological front and a convergence of improvements in the various steps involved in radiotherapy planning including scanning methods used to prepare for radiotherapy, computing techniques used to calculate radiotherapy, progressive upgrades in the radiotherapy delivery machines have led to quicker, safer, and easier delivery of SBRT. These have made SBRT an effective and safe treatment method, which has led to widespread adoption of this treatment technique across the world. Seminal papers on practical implementation of these treatment techniques in the context of a multicenter clinical trial [6] and detailed reports [7] on the methodology for achieving good quality assurance for these treatments have lent a high degree of confidence in the implementation of these techniques worldwide [1,8].

## 3. Current Applications

### 3.1. Non-Small-Cell Lung Cancer (NSCLC)

Surgery has long been the gold standard in treating early-stage lung cancer (for the purpose of this article defined as T1 or T2 N0M0 non-small-cell lung cancer as per AJCC v7.0). In most centers, cohorts of patients exist with significant medical comorbidities which render them unsafe for invasive surgical procedures. This group of patients presents a therapeutic challenge as the survival outcomes from alternative therapies like conventionally fractionated radiotherapy are usually inferior to surgical outcomes [9]. In a proportion of patients with pulmonary comorbidities, the comorbidity that prevented surgery from being a safe treatment option also precluded use of conventional radiotherapy. SBRT has proven to be a viable alternative to surgical treatment providing high local control rates and equivalent survival in matched patient cohorts [10,11]. 

The evidence and utility of this modality will be considered in three broad indications:

#### 3.1.1. Peripheral* Early-Stage Lung Cancers in Medically Inoperable Patients

*—defined as per the international association for the study of lung cancer (IASLC) as tumours within 2 cm of the proximal airways, mediastinal organs, and brachial plexus.

This indication is probably the most common reason for use of SBRT in early-stage lung cancer worldwide and perhaps the least controversial amongst lung cancer clinicians, but despite this randomized evidence for the two commonest modalities used to treat patients in this cohort, SBRT and conventional fractionated radiotherapy, is still lacking. Until recent trials addressing this very question were conducted, most of the results for patients treated with this modality were cohorts of non-randomized patients, the largest of which are summarized in Table 1. 

These cohorts consistently demonstrate the ability of SBRT to deliver a high degree of local control in the order of 90–95%. In retrospective comparisons with cohorts of patients treated with conventional radiotherapy (RT), SBRT has shown both improved survival and local control, a recent such comparison with a propensity matched cohort of 497 patients has demonstrated this, with a local failure rate of 34.1% with conventional radiotherapy and 13.6% with SBRT, corresponding to 3-year overall survival (OS) figures of 38.9% and 53.1%, respectively [24]. Most of the older retrospective comparisons [25] are biased against conventional radiotherapy due to the historical nature of the staging procedures employed and older treatment delivery techniques used for these treatments.

The SPACE trial [26] was a randomized phase 2 trial that recruited 102 medically inoperable Stage I non-small-cell lung cancer patients to either 66 Gy in 3 fractions SBRT in 1 week or 70 Gy in 35 fractions in 7 weeks conventional radiotherapy. Despite a larger proportion of T2 size tumours in the SBRT arm (47% vs. 25%), there was no statistically significant difference in survival between the two treatment arms in terms of overall survival at 3 years (54% in the SBRT arm vs. 59% in the conventional RT arm) or local control (86.4% vs. 85.7%). Toxicity was low in both arms but esophagitis was higher in the conventional RT arm (8% vs. 28%), and health-related quality of life was also worse in the conventional RT arm with dyspnea, chest pain, and cough being the main factors.

The authors concluded that, in the absence of any difference in survival, the lower toxicity and shorter treatment time of SBRT should make this the new standard of care in early-stage NSCLC.

The CHISEL trial [27] randomized 101 biopsy-proven inoperable Stage I NSCLC patients to either SBRT (54 Gy/3 fraction or 48 Gy/4 Fractions) or conventional RT (66 Gy/33 fractions or 50 Gy/20 fractions). Patients randomized to SBRT had improved freedom from local failure (Hazard ratio (HR) = 0.29, *p* = 0.002) and longer overall survival (HR = 0.51, *p* = 0.020). One patient in the SBRT arm experienced Grade 4 toxicity and 11 patients had grade 3 toxicity (2 CRT, 9 SBRT). This was the first randomized controlled trial to show superiority in overall survival for SBRT over conventional RT. This trial has only been presented in abstract form so far and a full peer-reviewed publication is awaited. The LUSTRE trial [28] is addressing a similar question in Canadian patients and has not reported yet. 

Overall, in peripheral early-stage NSCLC, SBRT achieves good local control with acceptable or superior overall survival in comparison to conventional RT, without excessive toxicity. In keeping with the available evidence, it is likely to remain the preferred treatment option in this cohort of patients. With less invasive surgical options with lower morbidity being developed, patients currently considered inoperable may be operable in the future, redefining the patient cohorts who are currently treated with SBRT. 

#### 3.1.2. Peripheral Early-Stage Lung Cancers in Medically Operable Patients

With the increasing implementation of SBRT for medically inoperable patients and increasing literature as documented in the above section of its utility in achieving high degrees of local control on par with surgical cohorts, the question of whether SBRT would offer the same degree of control as surgical resection in patients in whom surgery is still possible has been raised. This is especially relevant in the group of patients who have enough comorbidity to render them a high-risk candidate for surgery but not quite enough to rule out surgery as an option altogether. 

Retrospective comparisons of these two modalities are confounded by a fundamental difference in the patients having these two treatments-operability. Even in case-matched or propensity-matched analyses, the very factors that make a lung cancer patient inoperable have a profound effect on the survival. This was shown best in the meta-analysis carried out by Zheng et al. [29] which analyzed 63 SBRT and surgical studies, which included over 11,000 patients treated between 2000 and 2012 with SBRT or surgery. This analysis showed that lobectomy (LR) had improved survival in comparison to SBRT with a 5-year OS rate of 66.1% for LR vs. 41.2% for SBRT, with no statistically significant differences in local control (80% LR vs. 83.9% SBRT) or disease-free survival rates (74.8% LR vs. 65.8 SBRT) at 5 years. In the multivariate analysis when confounders like age and the percentage of operable patients were accounted for, the differences in overall survival were no longer statistically significant. The meta-analysis also found that the percentage of operable patients in the SBRT studies positively correlated with the OS, i.e., the higher the percentage of operable patients in the SBRT cohort, the better the survival, with a median of 12% operable patients, indicating that the vast majority of patients in the SBRT studies were inoperable. There was also a significant difference in the age of the patient cohorts recruited into surgical and SBRT trials, with SBRT trial participants being older (median age 74 vs. 66 years).

Other matched-pair analyses by Zhang et al. [30] comparing 864 matched patients across 6 studies have shown the superiority of surgery over SBRT in terms of 3-year OS (OR = 1.82 95% CI 1.38–2.40; *p* < 0.0001), with no difference in local control, disease-free survival or cancer-specific survival. Shirvani et al. [31] compared real world outcomes in 9093 older (median age 75) patients treated for early-stage NSCLC with lobectomy (LR), Sub lobar resection (SLR), and SBRT. They found that unadjusted mortality at 3 years was superior in patients undergoing LR (25%) as opposed to SLR (35.3%) or SBRT (45.1%). When adjusted with propensity score matching, the OS was equivalent in LR vs. SBRT (HR 1.01 95% CI 0.74–1.38). 

Due to the limitations of retrospective reviews, there have been a few prospective trials (ROSEL, STARS, ASCOG 4099, SABRTooth [32],) initiated to answer this clinical question but have closed early due to poor recruitment. The combined analyses of two of these trials [33], ROSEL and STARS, generated interesting results, with a 3-year OS of 95% for SBRT vs. 79% for surgery (HR 0.14 95% CI 0.017–1.190 *p* = 0.037). A total of 10% of SBRT patients had grade 3 toxicity with no grade 4 or 5 toxicity. This compared favorably to 4% grade 5, 4% grade 4, and 15% grade 3 toxicity seen in patients who had surgery. The authors concluded that SBRT is better tolerated and might lead to better overall survival compared to surgery. 

Further large phase 3 trials are required to adequately answer this question and some of these are in progress (VALOR, STABLE-MATES, POSTILV). Given the absence of equipoise amongst lung cancer clinicians regarding the two treatments available for this patient cohort, the ability to recruit to these trials may be as poor as the previous attempts at this, although the studies have actively tried to address this in innovative ways. In the absence of conclusive evidence either way, for most tumour boards and multidisciplinary team meetings, surgery remains the treatment of choice in medically operable patients. In patients in whom surgery is considered high risk, a joint discussion regarding SBRT and surgery between the surgeon, oncologist, and the patient should help guide the appropriate management strategy.

#### 3.1.3. Central Early-Stage Inoperable Lung Cancers

Central lung cancers (defined as per the IASLC as tumours within 2 cm of the proximal airways, mediastinal organs and brachial plexus [34]) are usually managed with surgery but due to location close to larger airways, the surgical approaches required are usually of a higher risk, i.e., pneumonectomy rather than lobectomy. This in turn leads to a larger impact on the patient’s respiratory/physiological reserve and therefore reduces the number of patients who will be fit enough to undergo this type of resection. Conventional fractionated RT is the alternative treatment in this setting but has the same potential problems as discussed in the section on peripheral tumours with potentially more toxicity especially with regards to the esophagus. There is a potential treatment niche for SBRT in this setting which is being explored. Most of the early and robust evidence regarding the utility of SBRT in lung cancer is based on peripheral lung tumours. Central lung cancers have a different therapeutic ratio for SBRT as treating more centrally located tumours with target volumes overlapping larger airways, vessels, and organs raises the potential for severe toxicity to these structures from high dose-per-fraction treatments. A phase II study by Timmerman et al. [35] highlighted these issues with a significant difference in freedom from severe (≥Grade 3) toxicity between central (54%) and peripheral (83%) lung cancers treated with the same dose (66 Gy in 3 fractions) of SBRT. Retrospective series [36,37] showed that risk-adapted strategies with lower biological equivalent dose (60 Gy in 8 fractions or 45–50 Gy in 5 fractions) and a larger number of fractions could potentially achieve the high degree of local control expected from SBRT without excessive toxicity but there were concerns that the degree of toxicity was an underestimate given the retrospective nature of these studies. 

Prospective studies addressing this cohort of patients have been reported showing a higher degree of grade 3 and above toxicity than that expected from SBRT of peripheral tumors. The Nordic HILUS trial [38] has reported a 28% incidence of grade 3 and above toxicity using a 56 Gy in 8 fraction regime for central tumours. The grade 5 (lethal) toxicity rate was high at 19% for tumours <1 cm from a main bronchus compared to 3% for tumours close to a lobar bronchus. Most of these were due to fatal hemoptysis with one case of fatal pneumonitis. The RTOG 0813 dose escalation trial [39] also reported a 4% incidence of fatal pulmonary hemorrhage although this was lower than the predicted 7.2% incidence for grade 5 toxicity. There was a trend for the higher grade (≥3) toxicities increasing with higher dose (21% for the highest dose cohort of 60 Gy in 5 fractions). The conclusion drawn by the authors was that severe toxicity could be mitigated by changes in dose fractionation. 

The EORTC LungTech trial [40] and the SUNSET trial [41] will further address this question when they report on the safety and efficacy in the cohort of central and ultra-central lung cancers. Until more toxicity data and longer-term follow-up from the previous trials are available, the SBRT of central inoperable tumours should be carried out ideally in the context of clinical trials or under controlled SBRT treatment programs with scrupulous radiotherapy quality assurance, conservative organ at risk (OAR) constraints to minimize toxicity, and regular rigorous follow-up to monitor toxicity. 

With lung cancer screening trials like NELSON [42] showing substantial reductions on mortality (26% at 10 years) from lung cancer, screening for early lung cancers in high-risk populations is likely to become reality in the future. With screen-detected early lung cancers, especially in the targeted group of patients with pre-existing airway disease and compromised lung function, techniques like SBRT and minimally invasive surgical techniques are likely to play an increasing role in improving survival in early lung cancer. 

### 3.2. Prostate Cancer

The incidence of prostate cancer is increasing with over 47,000 new cases reported in 2015, reflecting a 44% increase in rates since the 1990s [43]. Localized prostate cancer can be effectively managed with both surgery and conventionally fractionated radiotherapy showing good efficacy and equivalent survival but with differing toxicity. As 5- and 10-year survival is commonplace in this patient group where the median age of presentation is in the 7th decade and beyond, differences in toxicity become more relevant end points for most patients and surrogate markers of control like prostate specific antigen (PSA)/biochemical progression-free survival (bPFS) are used in studies as improvements in survival signal are likely to take over a decade of follow-up to determine. 

Surgical resection has traditionally resulted in increased genitourinary (GU) toxicity [44], whereas radiotherapy has resulted in more bowel (GI) toxicity. Like most cancers, there was a dose–response relationship for prostate cancer with higher doses of radiotherapy providing better cancer control and survival, at the cost of higher toxicity [45]. Improvements in radiotherapy delivery like image-guided intensity-modulated radiotherapy, driven by large-scale trials like the CHHiP trial [46], have resulted in a lower incidence of GI toxicity and an expanding interest in hypofractionation of radiotherapy in this setting. The greater understanding of the radiobiology of prostate cancer as a result of this trial and the hypothesized increased susceptibility to large dose-per-fraction radiotherapy regimes have led to the exploration of SBRT in the management of localized prostate cancer, as a form of extreme hypofractionation combined with high-quality image-guided radiotherapy to minimize toxicity. 

Interest in the use of robotic radiosurgery platforms in the treatment of prostate cancer drove the initial implementation of SBRT in this tumour setting. Some of the larger prospective studies are listed below in Table 2.

All the prospective series show excellent biochemical progression-free survival, with higher doses in the dose escalation studies showing higher rates of GU and GI toxicity. Most of this was early toxicity which resolved over a period of weeks to months. In the dose escalation studies, the higher doses (40 Gy in 5 fractions) did lead to a higher rate of late toxicity as well. As this occurred without any significant improvement in bPFS, the consensus was that a lower dose of SBRT would be adequate to achieve the high rates of control expected while more studies were required to refine the SBRT dose constraints for the organs at risk in this setting—mainly the rectum and urethra. As a result of these dose-finding studies, the doses used for prostate SBRT are in the range of 33–37 Gy/5 fractions.

Large-scale phase 3 trials are ongoing at present to define the role of SBRT in localized prostate cancer. The PACE trial [52] lead by Dr. VanAs at the Royal Marsden hospital, London, is a two-pronged multicenter phase 3 clinical trial comparing prostatectomy to prostate SBRT in PACE-A and conventional intensity-modulated radiotherapy (62 Gy in 20 fractions or 78 Gy in 39 fractions) to prostate SBRT (36.25 Gy in 5 fractions) in PACE-B. Recruitment for this trial is complete and results are eagerly awaited by the prostate oncology community to determine the role of SBRT in management of localized prostate cancer. 

### 3.3. Oligometastatic Disease

The concept of an oligometastatic state in cancer, where there was limited spread of the disease outside of the primary site, was first proposed in 1995 by Hellman & Weichselbaum [53]. The possibility of achieving a cure in disease considered incurable is an attractive proposition for most cancer clinicians and outside of a few very chemotherapy-sensitive malignancies, usually not achievable. With improving and increasing use of diagnostic imaging in follow-up settings, detection of small-volume early metastatic disease has become common and management paradigms for this are still in evolution. For a vast majority of cancers in the metastatic setting, definitive treatment is largely palliative systemic therapy, but in a small proportion of cancers in the oligometastatic setting (which is yet to be defined), more aggressive local treatment of the metastases could have a role to play in improving disease-free intervals and even achieving a cure. Surgery has been used in the setting of one or limited number of metastases restricted to on organ site like the liver or lung and there are published cohorts [54,55] of patients who have undergone surgical treatment for the metastases. Due to the lower levels of toxicity and high degree of local control offered by SBRT, there was considerable interest in the use of this modality in the oligometastatic setting. The maximum number of oligometastatic sites that a patient can have before the disease is considered disseminated is yet to be defined, but at present 1–5 sites are treated using SBRT in ongoing trials. 

A comprehensive review of non-randomized studies in the use of SBRT for oligometastases by Tree A. et al. [56] has demonstrated the safety and efficacy of this treatment. Local control rates are consistently high as expected at around 80%, but more interesting is an improvement in progression-free survival of around 20% at 2–5 years across these studies. This hints at changes in the natural history of the disease despite what was in essence local management. This was a feature also seen in the surgical resection series for oligometastases, indicating that in a proportion of patients, long-term control over the disease is a feasible end point to aim for. The doses used to treat oligometastatic disease are listed in Table 3.

To address this research question, two phase 2 trials were started, SABR-COMET [57] and CORE [58]. The SABR COMET trial recruited patients with up to five metastatic deposits, in three or less organs, which were metachronous (defined in the trial as occurring at least 3 months after the treatment designed to control the primary tumour) in the lung, liver, adrenal, spine/bone or brain and treatable with SBRT. Ninety-nine patients with oligometastatic breast, lung colorectal, or prostate cancer were randomized to either standard of care (SoC) or SBRT to all sites of metastases + SoC in a 2:1 ratio. Median overall survival was 28 months in the SoC arm and 41 months in the SBRT + SoC arm (*p* = 0.09). Median progression-free survival was 6 months in the SoC arm and 12 months in SBRT + SoC arm (*p* = 0.0001). Grade 2 or greater toxicity was 9% in the SoC arm and 30% in the SBRT + SoC Arm (*p* = 0.22) with fatigue, dyspnea, and pain being the commonest toxicity. Despite this, there were no differences in the quality of life scores in both arms. There were 3 treatment-related deaths in the SBRT arm from pneumonitis, lung abscess, and perforated gastric ulcer. The impression is that SBRT can prolong disease-free survival at the cost of toxicity and a phase 3 trial treating a larger number of patients is planned. It is notable that the toxicity reported in the trial is considerably higher than rates reported in most previous studies in this context. 

The CORE trial is addressing a similar question in oligometastatic breast cancer, NSCLC, and prostate cancer patients with up to 3 metastases in a maximum of 2 organs sites defined as oligometastases. In this trial, the definition for metachronous disease was >6 months from definitive treatment of the primary tumour in breast and prostate histology and >4 months for NSCLC. Metastases in the lung, bone/spine, liver, adrenal and lymph nodes are treated on this trial with rigorous centralized radiotherapy quality assurance of both clinicians and institutions. The trial is actively recruiting and due to complete recruitment in 2019.

Both these trials specifically excluded synchronous oligometastases. The assumption was that patients with synchronous metastases, with a larger disease load, have poorer outcomes compared to metachronous disease (and presumably to avoid the confounding factors of the extent of treatment of the primary tumour). This may not be a valid assumption as demonstrated by Fleckenstein et al. [59], where radical treatment of both primary non-small-cell lung cancer and oligometastases in both settings (synchronous and metachronous) had equally good survival outcomes. In keeping with this line of reasoning, the SARON trial [60], a phase 3 trial of platinum agent-based chemotherapy followed by either radical dose radiotherapy plus SBRT to oligometastases or standard of care treatment in oligometastatic NSCLC, is currently recruiting.

The treatment of oligometastases has raised the question of the utility of SBRT in controlling ‘oligoprogression’. The hypothesis is that the cancer clone/stem cell containing the resistance mechanism for the ongoing treatment (e.g. hormone antagonist therapy) has arisen in one of the metastases against a background of stable or responding disease and that ablating this source of resistant disease could prolong survival. The HALT study [61] is looking at the utility of SBRT in the context of oncogene-addicted lung cancer on tyrosine kinase inhibitor (TKI) treatments, looking to recruit 110 patients randomizing between SBRT of up to 3 sites of oligoprogression +TKI vs. continuing TKI alone. This approach could potentially be expanded into other settings where a ‘maintenance’ systemic therapy is keeping the majority of the metastatic disease at bay, e.g., hormone-responsive metastatic malignancies like breast and prostate cancer on endocrine therapy.

SBRT has a high rate of achieving local control in oligometastatic disease in sites like the spine where surgical intervention can be a high-risk undertaking. The use of SBRT for treatment of spinal oligometastatic disease was the second most frequent indication (after primary lung cancer) for use of SBRT in the SEER database. The concern about using higher doses of radiotherapy for control of spine metastases largely centered on the relatively low radiation tolerance of the spinal cord. This is especially a problem when the segment of spine has been previously treated with radiotherapy either for the same metastases or to an adjacent tumour, e.g., in the case of paravertebral lung tumours. With the use of magnetic resonance imaging (MRI) scans of the spine, specifically done for the radiotherapy planning process, the spinal cord and cauda equina are better visualized. This imaging was combined (co-registered) with the treatment planning CT imaging to achieve delineation of the spinal cord accurately on the radiotherapy treatment planning software in preparation for SBRT planning. International consensus guidelines on delineation of the target volumes have been published [62] and have led to standardization of the volumes treated with Spine SBRT in most centers. In addition to these improvements, the use of rigorous image guidance which has become the hallmark of SBRT treatments, to accurately correct millimeter changes in spine or patient position during delivery of the treatment, allowed SBRT to minimize the risk of radiation myelopathy by controlling the dose delivered to the spinal cord and other organs at risk (as shown in Figure 1a). 

Several large case series and prospective studies (Table 4) have demonstrated the ability of spinal SBRT to achieve a high degree of local control, optimize pain control, and improve quality of life. These improvements in local control are achieved with minimal acute toxicity as shown in the absence of any grade 4 toxicity and minimal grade 3 toxicity. However, late effects, which were uncommon with conventional radiotherapy like vertebral compression fractures (5%), are seen more frequently with SBRT (11–30%) [63]. The potential predictors of toxicity are being recognized in prospective SBRT cohorts and scoring systems using these predictors have been developed to identify the patients with risk of instability of the spine who may benefit from surgical intervention [64]. The potential route to minimizing these toxicities could be to use a multidisciplinary approach. By identifying patients at risk of developing spinal fractures and instability, using validated scoring systems and using minimally invasive spinal interventions like kyphoplasty (before or) after SBRT, spinal stability can be achieved without compromising on local tumour control [65]. 

SBRT is playing an increasingly important role in achieving local control in oligometastatic disease. Ongoing trials will inform us as to whether this expanding role is achievable with acceptable levels of toxicity and clarify the role of SBRT in influencing survival. Combining SBRT with other treatments like surgery and immunotherapy to leverage the strengths of each treatment modality without exacerbating toxicity will be the target of future research in this area.

### 3.4. Other Indications

#### 3.4.1. Primary Hepatic Cancer

Hepatocellular carcinoma HCC usually arises in patients with liver cirrhosis, while a small proportion of these patients can be curatively treated with liver transplantation for a majority radiofrequency ablation, trans-arterial chemo-embolization (TACE) are the available local treatment options to serve as a disease control bridge till a transplant becomes available. SBRT can be another option to use where technical limitations like proximity to a large vessel, large tumour, etc. render other local treatment options non feasible. SBRT for HCC has been proven [72] to serve as a bridge to transplant treatment with a median OS of 17 months and local control rate of 85%.

#### 3.4.2. Primary Inoperable Kidney Cancer

SABR has a role to play in the management of medically inoperable primary kidney cancer. Pooled analysis of cases treated with SBRT [73] have shown a local control rate of 97% and PFS of 65% at 4 years coupled with a low toxicity rate of 1.3% grade 3 or greater toxicity. The deterioration in estimated glomerular filtration rate (EGFR) was also at an acceptable median of 10%. 

#### 3.4.3. Re-Irradiation

The use of high-quality image guidance combined with tight margins on the treatment target volume make SBRT an attractive method of re-irradiation, where toxicity concerns of organs at risk close to a previously treated area are determinants of dose. SBRT has been successfully used to re-treat spinal tumours [74], pelvic relapses [75] and head and neck squamous cancer [76] recurrences with acceptable levels of toxicity and high local control. 

## 4. Future Directions

Current trials are likely to inform us on what the role of SBRT will be in operable lung cancer, prostate cancer and oligometastatic disease. Given the high local control rate and low toxicity in comparison to other available treatment modalities combinations with systemic agents, especially immunotherapy, are promising. The ability of SBRT to recruit immunostimulatory CD8 T lymphocytes into tumour cells and PDL-mediated abscopal tumour cell kill are exciting research avenues to explore and are the target of several trials combining immunotherapy with SABR or iSABR [77].

## 5. Conclusions

SBRT offers a noninvasive treatment option with a high degree of local control with manageable toxicity in treating most tumour sites in the primary and metastatic setting. A growing body of evidence supporting its utility as an alternative to surgery in various circumstances is emerging. Prospective trial evidence to accurately assess the toxicity in patient cohorts and rigorous quality assurance with regards to its planning and delivery are essential if this modality is to be optimally used in the wider radiation oncology community. 

## Figures and Tables

**Table 1:** Primary Non-Small-Cell Lung Cancer SBRT cohorts.

Author	No. of Patients.	Local Control	Overall Survival (OS)
Nagata et al. 2005 [12]	45	95% at 5 years	83% at 5 years
Onishi et al. 2007 [13]	257	86% at 3 years	56% at 3 years
Lagerwaard et al. 2008 [14]	206	93% at 2 years	64% at 2 years
Haasbeek et al. 2010 [15]	193	89.3% at 3 years	45.1% at 3 years
Bongers et al. 2011 [16]	500	90.4% at 3 years	53.1% at 3 years
Palma et al. 2012 [17]	176	89% at 3 years	47% at 3 years
Senthi et al. 2012 [18]	676	10.5% LRR at 5 years	Not available
Chang et al. 2012 [19]	130	98.5% at 2 years	65.3% at 3 years
Gillespie et al. 2015 [20]	320	95% at 2 years	64.25% at 2 years
Murray et al. 2015 [21]	273	95.7% at 3 years	38.6% at 3 years
Shaverdian et al. 2016 [22]	118	97% at 3 years	77% at 3 years
Chiang et al. 2016 [23]	192	89.3% at 3 years	72.4% at 3 years

**Table 2:** Prostate SBRT prospective cohorts.

Author	No. of Patients	Dose/No. of Fractions(#)	bPFS *	Early Toxicity (≥Grade2)	Late Toxicity (≥Grade 2)
Bolzicco et al. 2013 [47]	100	35 Gy/5#	94.4% at 3 years	GU 12%, GI 18%	GU 3%, GI 1%
Meier et al. 2016 [48]	309	36.25–40 Gy/5#	97.1% at 5 years	GU 26%, GI 2.8%	GU 14%, GI 2%
Loblaw et al. 2017 [49]	114	35–40 Gy/5#	97.3% at 5 years	Not reported	Not reported
Helou et al. 2017 [50]	259	35–40 Gy/5#	Not reported	Not reported	GU 34%, GI 14%
Fuller et al. 2017 [51]	259	38 Gy/4#	100% at 5 years	Not reported	GU 13.7%, GI 4.5%

* biochemical progression-free survival.

**Table 3:** SBRT doses used in the treatment of Oligometastatic disease.

Site of Metastases	Dose (Gy)	No. of Fractions
Peripheral lung metastasis not adjacent to chest wall	54–60	3
Peripheral lung metastases adjacent to chest wall	55	5
Central lung metastases	60	8
Adrenal metastases	36	3
Liver metastases	30–60	3–10
Lymph node	30	3
Spine metastases	24 or 27	2 or 3
Bone (non-spine) metastases	30–45	3

**Table 4:** Spine SBRT Cohorts.

Study (Year)	No. of Patients	Dose/No. of Fractions(#)	Local Control	Pain Control	≥Grade 3 Toxicities
Chang et al. 2009 [66]	129	16–39 Gy/1–5#	69% at 3 years	91%	No toxicity
Schipani et al. 2011 [67]	124	18 Gy/1#	92% at 3 years	92%	No toxicity
Wang et al. 2012 [68]	149	27–30 Gy/3#	72% at 2 years	86%	Acute 9% (pain, GI, fatigue)
Guckenburger et al. 2014 [69]	301	Median 24 Gy/3#	84% at 2 years	Not reported	Acute 0.01% (pain)
Bishop et al. 2015 [70]	285	18–27 Gy/1–3#	82% at 3 years	Not reported	Not reported
Bernard et al. 2017 [71]	127	Median 27 Gy/3#	76% at 2 years	Not reported	6.75% Spine fracture
